# Functionality of Pea-Grass Carp Co-Precipitated Dual-Protein as Affected by Extraction pH

**DOI:** 10.3390/foods11193136

**Published:** 2022-10-09

**Authors:** Xiaohu Zhou, Chaohua Zhang, Liangzhong Zhao, Wenhong Cao, Chunxia Zhou, Xin Xie, YuLian Chen

**Affiliations:** 1 College of Food Science and Technology, Guangdong Ocean University, Zhanjiang 524088, China; 2 College of Food and Chemical Engineering, Shaoyang University, Shaoyang 422000, China; 3 Guangdong Provincial Key Laboratory of Aquatic Products Processing and Safety, Zhanjiang 524088, China; 4 Hunan Provincial Key Laboratory of Soybean Products Processing and Safety Control, Shaoyang 422000, China; 5 Collaborative Innovation Center of Seafood Deep Processing, Dalian Polytechnic University, Dalian 116034, China

**Keywords:** pea, co-precipitated dual-protein, blended dual-protein, plant protein, grass carp, surface hydrophobicity, emulsifying properties, gel properties, mixed protein

## Abstract

Isoelectric solubilisation/co-precipitation (ISP) has been proven to be a better method than blending for preparing plant–animal dual-proteins, which can achieve synergies in the functional properties of heterologous proteins. This paper aims to investigate the effect of extraction pH on the functional properties of co-precipitated dual-protein. The basic composition, subunit composition, solubility, surface hydrophobicity, emulsification and gel properties of co-precipitated dual-protein (Co) prepared from pea and grass carp with pH (2.0, 3.0, 9.0, 10.0 and 11.0) were analysed in this study using ISP. The results showed that the functional properties of Co (Co9, Co10, Co11) prepared by alkali extraction were generally better than those prepared by acid extraction (Co2, Co3). Among them, Co10 has the highest vicilin/legumin α + β value and solubility, while having the lowest surface hydrophobicity, making its emulsification and gel properties superior to other extraction pH values. This study provides an important method reference for preparing plant-animal Co with exceptional functional properties.

## 1. Introduction

Currently, there is an increasing demand for high-quality animal proteins such as meat, eggs and milk, but high dietary patterns in animal protein can lead to excessive greenhouse gas emissions and cannot be produced sustainably [1]. Plant protein is more economical and environmentally friendly, but it is challenging to meet the needs of essential amino acids completely, making a vegan diet unsuitable for teenagers and other consumer groups [2]. Because mixed plant and animal proteins can achieve ‘protein complementarity’ and better meet the needs of nutrition and environmental protection, the preparation of animal and plant double proteins has become a research focal point [3].

**Figure 1:**
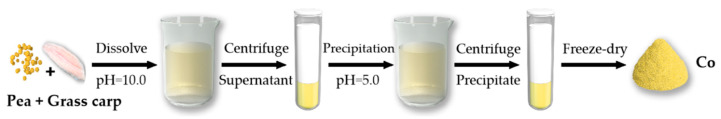
Schematic diagram of the preparation of co-precipitated dual-protein (Co).

Dual-proteins can be divided into blended dual-protein (BL) and co-precipitated dual-proteins (Co) according to the preparation method [4]. BL refers to a protein created by blending heterologous protein isolates directly. Co is prepared by ISP. The process of Co preparation is shown in Figure 1. We previously discovered that the functional properties of pea-grass carp Co, such as dissolution, emulsification and gel, are significantly better than those of BL; moreover, pea-grass carp Co had better functional properties than a single protein under some conditions, with heterologous proteins exhibiting synergistic effects [5,6]. A study confirmed that the advantage of the ISP method is that proteins from different sources dissolve and precipitate in the same dispersion system, promoting the interaction between heteroproteins, generating disulfide bonds and changing protein subunit composition, surface charge, solubility and *H*_0_; consequently, functional properties are effectively improved [7]. Subsequently, several studies have found that proteins extracted at different pH levels have different functional properties [8]; for example, proteins extracted under alkaline pH conditions have better nutritional properties [9], heat-induced gel properties and sensory properties [10]. Furthermore, evidence suggests that acidic solubilisation results in slightly higher protein recovery [11]. Evidently, the extraction of pH has a significant impact on the protein’s function.

Compared to soybean, pea *(Pisum sativum* L.) has almost no sensitisation and transgenic source risk, which has recently drawn significant attention to pea protein [12]. Peas are one of the world’s most widely planted and consumed legumes, with a global output of 14.64 million tonnes in 2020 [13]. Peas contain 20–30% protein, 65–80% globulin and 10–20% albumin. They are rich in lysine and have a high nutritional value but lack methionine. The isoelectric point of pea protein is about 4.5, which shows good emulsification and gel properties when applied in processing [14]. Grass carp (*Ctenopharyngodon idellus*) is the most productive freshwater aquaculture product in China, with an output of 5.57 million tonnes in 2020 [15]. Fish protein has a high nutritional value, and its methionine content is second only to that of eggs [16]. The protein isoelectric point of grass carp muscle is about 5.5 and has a high salt-soluble protein content and gel strength. However, due to its poor thermal stability, modifying or mixing it with other proteins is required during processing [17,18]. Therefore, it can be concluded that the ‘protein complementarity’ of a single protein can be realised by combining high-quality and low-cost pea protein and grass carp protein, effectively improving nutritional and functional properties.

Previously, we found that when ISP was used to prepare Co of soybean-tilapia (protein mass ratios of 3:1, 2:1, 1:1, 1:2 and 1:3, respectively), the solubility and nutritional value of “1:1” had certain advantages. We also found that the solubility of Co prepared by acid or alkali methods were significantly different, but the influence of the extraction pH on the protein subunit composition, emulsification properties and gel properties was not discussed [19]. In this study, Co with a mass ratio of pea protein to grass carp protein of 1∶1 was selected as the research object, and the effects of the extraction pH (2.0, 3.0, 9.0, 10.0, 11.0) on the subunit composition, solubility, surface hydrophobicity, emulsifying properties and gel properties of the Co were investigated in order to identify the optimal parameters for the production of Co with excellent functional properties. The purpose of this paper is to provide a theoretical basis for a broadened application of Co in the food industry.

## 2. Materials and Methods

### 2.1. Materials

Pea flour was provided by the Foshan Jinnuoyi Processing Factory of Agricultural Products Co., Ltd. (Foshan, China); it contained 21.12% protein. The grass carp was purchased at a seafood market in Zhanjiang City, China. The gills, heads, tails, bones and blood were all removed, leaving only the white muscles on the back, which were retained for storage at −20 °C; they also contained 21.12% protein. MD1477 dialysis bags (regenerated cellulose, 8000–14000) were purchased from Shanghai Yuanye Biotechnology Co., Ltd. (Shanghai, China). The SDS fast electrophoresis kit was provided by Beyotime Co., Ltd. (Shanghai, China). The Lowry method protein content determination kit was provided by Shanghai Lida Co., Ltd. (Shanghai, China). ANS fluorescent probe was provided by Aladdin Technology Co., Ltd.

### 2.2. Preparation of Co

Co was prepared using the ISP method (Figure 1). Pea flour and fish mince were mixed at a certain mass ratio and then mixed with 4 °C deionised water at a ratio of 1:9 (W/V). The pH was adjusted to 2.0, 3.0, 9.0, 10.0 and 11.0 with 1 M HCL or NaOH and dissolved for 10 min, followed by centrifugation at 10,000 rpm for 10 min (J-26sxp; Avanti, Beckman, Indianapolis, IN, USA)). The sediment was removed, and 1 M NaOH or HCl was added to the supernatant. Stirring was continued for 20 min, the pH was adjusted to 5.0 and centrifugation was repeated. At this time, the precipitation was washed with deionised water, and the pH value was adjusted to 7.0. Then, dialysis was performed for 72 h. Upon freeze-drying for 48 h, Co2, Co3, Co9, Co10 and Co11 were obtained.

To ensure an analogous comparison, the mass ratio of pea protein and grass carp protein in Co was maintained at 1:1 for this study. The protein recovery ratio in the sediment was calculated by dissolving pea flour and fish mince at pH 2.0, 3.0, 9.0, 10.0 and 11.0, respectively, and precipitation was performed at pH 5.0; the supernatant was removed after centrifugation and the yield of protein in the sediment was calculated. Then, the following formula was used to calculate the required raw material for the mass ratio (i.e., 1:1). When preparing Co with different extraction pH levels, the 1:1 ratio Co can only be obtained according to the corresponding raw material mass ratio.

Pea flour mass (g) × protein content of pea flour (%) × protein recovery ratio (%) = Fish mince mass (g) × protein content of fish mince (%) × protein recovery ratio (%)(1)

### 2.3. Solubility

A 0.5% (*w*/*v*) protein solution of Co was prepared and stirred at a constant speed for 30 min. The pH was then adjusted to 4.0–11.0 and the solution was stirred for 30 min, followed by centrifugation (10,000× *g*, 4 °C, 120 min). The protein content in the supernatant was determined at 750 nm using the Lowry method. The solubility calculation formula was as follows:

Solubility (%) = C/C_0_ × 100%, (2)

In this equation, protein content after centrifugation (mg) is represented by C, while protein content before centrifugation (mg) is denotes by C_0_.

### 2.4. Non-Reduced SDS-PAGE

The original protein compositions of Co were characterised by non-reduced sodium dodecyl sulfate-polyacrylamide gel electrophoresis (non-reduced SDS-PAGE), as follows. Solutions of 1% protein concentration were prepared from the known solubility of Co in Section 2.3. The volume fraction of concentrated gel (upper gel) was 5%, while the volume fraction of separated gel (lower gel) was 12%. No reducing agent dithiothreitol (DTT) was added; the protein marker was 16–270 kDa. Protein band abundance was expressed by relative optical density (%), measured using Image LabTM V4.0 software (Bio-Rad Laboratories, Hercules, CA, USA).

### 2.5. Colour

A colourimeter (NS800, Shenzhen, Guangdong, CNN) was used for colourimetry of Co, which was represented by the L* value, a* value and b* value. The following formula was used to calculate the whiteness [20].

(3) $$\mathrm{Whiteness}=100-\sqrt{(100-L*{)}^{2}+{a*}^{2}+b{*}^{2}}$$

### 2.6. Surface Hydrophobicity (H_0_)

In accordance with Haskard [21], the ANS fluorescent probe was used to determine the *H*_0_ value. Co was formulated as a 0.5% protein solution in 50 mL and added to a 10 mmol/L phosphate buffer (pH 7.0). After stirring for 1 h, centrifugation was performed at 7500× *g* for 30 min. The protein concentration of the supernatant was determined by the Lowry method and then diluted successively with the same supernatant (concentration 0.005–0.5 mol/mL). Then, 4 mL of each concentration solution was mixed with 40 μL ANS solution of 8 mmol/L (prepared with the same phosphate buffer) and kept standing for 5 min after oscillation. Afterwards, the spectrophotometer was used to determine the fluorescence intensity of the sample. The excitation wavelength was set to λex = 370 nm, the emission wavelength was set to λem = 490 nm and the crack was set to 5 nm. The fluorescence intensity was plotted on the *Y*-axis, the protein concentration on the *X*-axis and the slope of the initial section was taken as the *H*_0_ of Co.

### 2.7. Emulsifying Property

#### 2.7.1. Preparation of Emulsion

Co was prepared as a 1.0% protein solution, mixed with soybean oil at 9:1 (*v*/*v*). The protein solution was treated with a T18 homogeneous shear machine (IKA Laboratory Equipment, Staufen, Germany)) for 1 min at 9000 rpm and 15,000 rpm, and the emulsion was stored at 4 °C.

#### 2.7.2. Emulsifying Activity Index and Emulsifying Stability Index

The EAI and ESI of the proteins were measured via turbidimetry [22]. For 30 min, 0.5% protein solution was prepared and dissolved. In total, 6 mL of protein solution was mixed with 2 mL of soybean oil and treated with a homogeneous shear machine for 1 min (12,000 rpm). Then, 20 μL of emulsion was taken from the bottom of the container and dispersed into 4 mL of 0.1% SDS solution. The absorbance was measured at 500 nm (A_0_). After 10 min, 20 μL of emulsion was taken from the bottom of the container again and dispersed into 4 mL of 0.1% SDS solution. At 500 nm, the absorbance was measured (A_10_). The same SDS solution was used as a blank control. The EAI and ESI were calculated as follows:

(4) $$\mathrm{EAI}\;=\frac{2\times 2.303\times A0\times D}{C\times \varphi \times 10^{4}}(m^{2}/g)$$

(5) $$\mathrm{ESI}\;=\frac{A_{0}}{A_{0}-A_{10}}\times \Delta t\;(\min)$$

where D refers to the dilution factor (200), C denotes the protein concentration (5 mg/mL), φ is the volume fraction of soybean oil in emulsion (0.25), L represents the optical cuvette path length (1 cm) and Δt indicates the measurement interval (10 min).

#### 2.7.3. Rheological Properties of Emulsion

The rheological properties of the emulsion were measured by a rheometer (DHR-2 Shear Rheometer, New Castle, DE, USA). Set shear rate range of 0.1–200 s^−1^, select a 40 mm type plate, control the temperature to 25 ℃ and determine the apparent viscosity of the emulsion.

#### 2.7.4. Microstructure of Emulsion

A total of 5 μL of emulsion at 4 °C was placed on a glass slide and covered with a cover glass. The emulsion droplets were observed with a microscope (CKX41, Olympus, Tokyo, Japan) under 400 magnification and photos were taken.

### 2.8. Gel Property

#### 2.8.1. Preparation of Heat-Induced Gel

Co was prepared as a protein solution with a concentration of 12% (*w*/*w*) and placed in a constant-temperature water bath. After heating to 90 °C at a rate of 1/min, it was kept for 30 min and then quickly cooled. After 6 h of standing at 4 °C, the gel sample was ready to use.

#### 2.8.2. Water-Holding Capacity (WHC)

The gel was cut into small pieces with a length, width and height of about 5 mm, respectively: the gel mass of M_1_(about 3 g) was weighed. The gel was wrapped in two layers of filter paper and centrifuged for 10 min at 10,000× *g*. The gel was removed and weighed, and its mass after centrifugation was M_2_. The WHC of the gel is calculated as follows:

(6) WHC (%)=M2/M1×100

#### 2.8.3. Hardness and Springiness

The hardness and springiness of the Co heat-induced gel samples were measured using a texture analyser. The following are the main parameters: P35 cylindrical flat bottom probe; sample flat surface height of 20 mm; test front, middle and final speeds of 4.0 mm/s, 3.0 mm/s and 4.0 mm/s; descending distance of 40%; compression time of 5 S and triggering force of 5 g.

#### 2.8.4. Microstructure of Gel

The microstructure of the gel samples was observed using a scanning electron microscope. The gel was cut into small pieces with a length, width and height of 3 mm × 2 mm × 2 mm, respectively, and placed in a container with a lid. The gel was soaked in phosphate buffer containing 2.5% pH 7.2 glutaraldehyde for more than 8 h at 4 °C before being washed three times for 10 min each with 0.1 mol/mL pH 7.2 phosphate buffer. Elution was repeated three times for a total of 10 min with 100% anhydrous ethanol. The dehydrated samples were degreased with the proper amount of trichloromethane for 1 h and then replaced with a 1:1 mixture of tert-butanol and ethanol for 15 min, followed by 100% tert-butanol for 20 min before being freeze-dried. The surface morphology of the sample was observed by scanning electron microscope (Gemini300, Zeiss, Oberkochen, Germany) with a 5000× field of vision.

### 2.9. Statistical Analysis

IBM SPSS software (Version 26.0; SPSS Inc., Chicago, IL, USA) was used for data analysis. ANOVA and Duncan’s multiple range test (*p* < 0.05) were used to determine the statistical difference between the two groups. Each measurement was repeated three times, and the results were expressed as mean ± standard deviation (SD). 

## 3. Results and Discussion

### 3.1. Protein Composition Analysis

**Figure 2:**
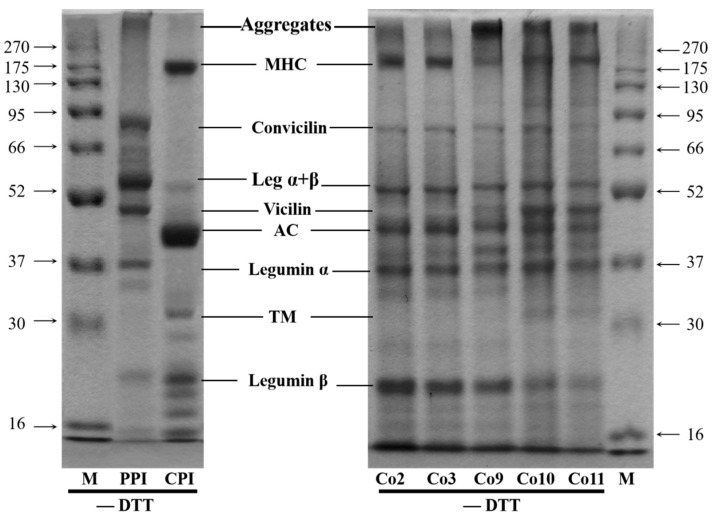
Non-reduced electrophoretic map of PPI, CPI and Co prepared under different extraction pH conditions.

Non-reduced SDS-PAGE electrophoresis can reflect the original composition of protein in the mixed protein [23]. The protein compositions of Co2, Co3, Co9, Co10 and Co11 are shown in Figure 2 and Table 1. To make the composition patterns of pea protein and grass carp protein more intuitive, PPI and CPI are also included in Figure 2. All Co involve soluble aggregates, myosin heavy chain (MHC, ~200 kDa), actins (AC, ~42 kDa), tropomyosin (TM, ~34 kDa), convicilin (~70 kDa), legumin α + β (~62 kDa), vicilin (~50 kDa), legumin α (~38 kDa) and legumin β (~22 kDa), consistent with the literature [24,25]. It can be seen that due to the close molecular weight or interaction, some protein bands of the two heterologous proteins are bound to overlap. ‘Blank’ appeared in the viclin electrophoretic bands of Co2 and Co3, and the recovery ratio of vicilin was high when pea protein was extracted in acid solution alone, and the corresponding electrophoretic bands were apparent [26]. By observing the relative positions of legumin α + β and AC bands of five kinds of Co in the electrophoretogram, it is speculated that the viclin of Co2 and Co3 may have some combination with AC during co-precipitation (the optical density is 16.72% and 18.47%). The TM band was not visible in the electrophoretogram of Co2 and Co3, and its optical density was zero in the analysis software, while it was clear in the electrophoretogram of Co10 and Co11 (optical density 4.60% and 2.30%, respectively), because some fish proteins, such as TM, were more denaturated in acid solution and were better suited for alkali solution recovery [27]. In general, the recovered proteins were more complete under alkaline conditions. Simultaneously, all Co could effectively synthesise the main proteins from pea and grass carp, which could be used for further experiments.

The main characteristic of non-reduction electrophoresis is that the disulfide reducing agent (DTT) is not mixed into the loading buffer, allowing the sample to retain all soluble aggregates formed by disulfide covalent crosslinking. According to research reports and preliminary experiments, the non-reduced electrophoretic patterns of pea protein [28] and grass carp protein [29] prepared by the ISP method had fewer top bands, indicating that there were fewer soluble aggregates with a high molecular weight. The top of the electrophoretogram of the five kinds of Co, especially Co9, Co10 and Co11, which evidently had aggregates, indicating that a certain degree of covalent cross-linking occurred between pea and grass carp protein during co-precipitation, resulting in soluble aggregates. Table 1 shows that soluble aggregates of Co9, Co10 and Co11 are greater than those of Co2 and Co3, indicating that the alkali method can generate more disulfide bonds. Studies have shown that the formation of disulfide bonds is conducive to increasing the flexibility of protein structure, thereby enhancing its emulsifying ability [30]. At the same time, disulfide bonds play an important role in stabilising the network structure of proteins during thermal gelation [31], suggesting that alkaline solution (Co9 Co10 Co11) is more advantageous than acid solution (Co2 Co3) in terms of the functional properties of proteins.

Notably, when legumin protein is present, the value of vicilin/legumin α + β greatly affects the functional properties of the protein. Vicilin is a protein with a more flexible structure and has good solubility, interface stability and gelation ability [32]. Many studies have confirmed the positive correlation between the value of vicilin/legumin α + β and the functional properties of dissolution, emulsification and gelation [33,34]. The quantitative analysis of the relative optical density of protein bands showed that Co10 was the highest (265.16%) among the five kinds of Co; therefore, the analysis of protein composition can be an important indication that Co10 has certain advantages in functional properties.

### 3.2. Colour

The colour of a protein can affect its application in food. The colour comparison test can objectively evaluate the colour of protein samples. L* denotes brightness; a* positive value represents redness; the negative value represents greenness; b* positive value represents yellowness and the negative value represents blueness. The analysis results of the colour of Co flour under different extraction pH conditions are shown in Table 2. The brightness values of Co11 and Co10 were 74.21 and 71.23, respectively, which were higher than those of other proteins. The whiteness of Co10 was slightly less than that of Co11 (*p <* 0.05) but significantly higher than Co2, Co3 and Co9 (*p <* 0.05). This is because dissolution under higher alkaline conditions will expand the structure of haemoglobin in fish protein, leading to oxidative denaturation and loss of heme [35], resulting in down-regulation of redness and improvement of brightness and whiteness. It is noteworthy that the whiteness value of Co9 is only 62.07, while the b* value is the highest (*p <* 0.05), which may be due to the high recovery rate of plant pigment of pea protein at pH 9.0 and the significant increase of yellow colour. Overall, the Co9 colour is not ideal and may have limited applications. In addition, Co10 and Co11 have similar colour properties, so they have potential applications in dairy products and surimi products with high whiteness requirements.

### 3.3. Solubility

**Figure 3:**
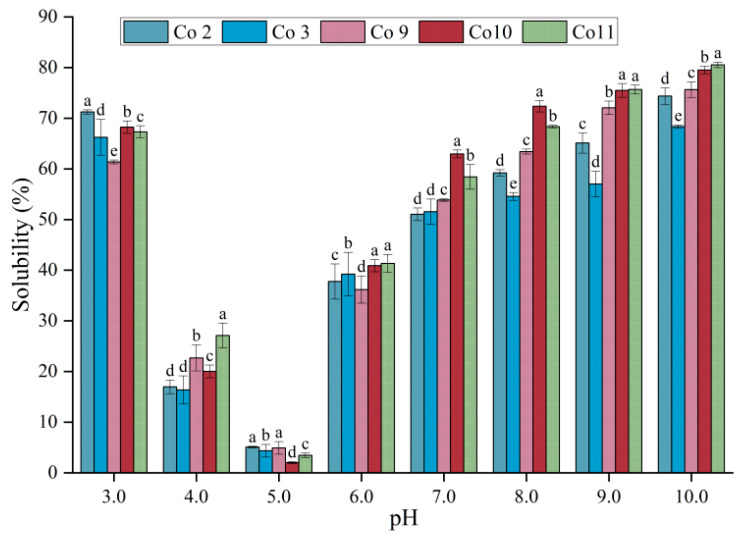
Solubility of Co prepared under different extraction pH conditions. Note: Different letters with the same pH indicate that there is a significant difference between samples (*p* < 0.05).

Solubility is one of the most important functional properties of food protein. It can be used to evaluate the potential use of protein in food and determine its emulsifying and gelling ability [36]. As can be seen in Figure 3, the solubility of the five kinds of Co is significantly correlated with pH, and the solubility presents a U-shaped curve. The solubility is minimum at pH 5.0, which is similar to the results of the dissolution curve of the soy-tilapia dual-protein [19] in previous studies. The main reason for this is that the isoelectric point of pea globulin is 4.5 [37], and that of grass carp protein is 5.5 [38]. When a protein approaches the isoelectric point, its net charge reaches zero, hydration is at its weakest and electrostatic repulsion between proteins disappears. Furthermore, at pH 5.0, the charge of pea protein is opposite that of grass carp protein, resulting in electrostatic attraction. Simultaneously, as the protein structure unfolds, the hydrophobic groups become exposed, resulting in hydrophobic aggregation and sedimentation, and the solubility is at its lowest. When the acidic pH ranges away from the isoelectric point, the negative charge of the carboxyl group of acidic amino acids is neutralised, leaving the protein with a net positive charge. Under alkaline conditions, the basic groups of some amino acids tend to deprotonate, resulting in a net negative charge of proteins, thus enhancing protein hydration and improving protein solubility [39].

Under the same pH, the solubility of the five kinds of Co also varies greatly. Under acidic conditions, such as extremely acidic pH 3.0, the solubility of Co2 is the highest (*p <* 0.05). Co11 solubility is highest under alkaline pH 10.0 (*p <* 0.05). When the pH is 6.0–9.0, the solubility of Co10 and Co11 is high, indicating that alkali extraction is superior to acid extraction, which is consistent with the results of soybean-tilapia co-precipitated dual-protein [40]. The solubility of Co10 was the highest at pH 7.0 and 8.0 (*p <* 0.05), reaching 62.95% and 72.36%, respectively, higher than commercial pea protein isolate [41], soybean protein isolate [42] and grass carp protein [29], indicating that the solubility of the dual-protein is synergistic. The higher value of vicilin/legumin α + β may be responsible for the higher solubility of Co10 and Co11. It can be seen that different extraction conditions may change solubility by changing the protein composition. Except for fermented food, the pH of most high-protein food systems is 6.0–8.0, so the high solubility of Co10 suggests its potential application in the food industry.

### 3.4. Surface Hydrophobicity (H_0_)

**Figure 4:**
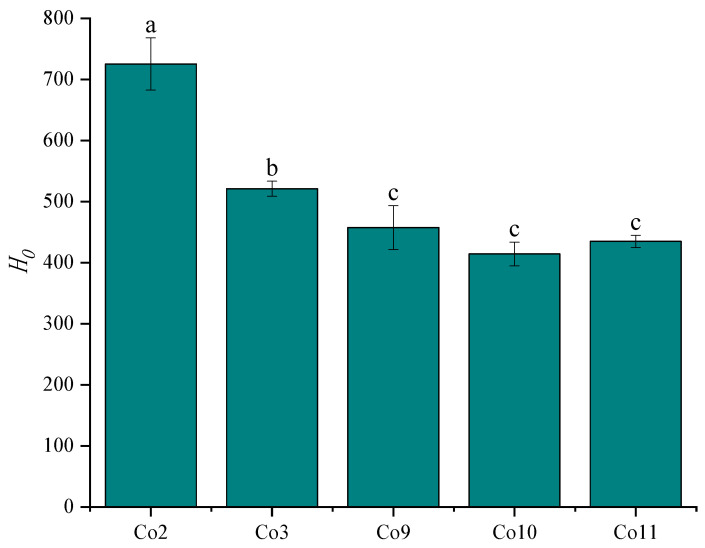
Surface hydrophobicity of Co prepared under different extraction pH conditions. Note: Different letters indicate that there is a significant difference between samples (*p* < 0.05).

Hydrophobic interaction is the main force of protein–water and protein–protein interaction. Proper surface hydrophobicity is essential for the formation of functional properties, such as protein solubility, gas-liquid and oil-water interface activity, thermal stability and gel-forming ability. The hydrophobicity of a protein can be characterised by the amount of hydrophobic amino acid residues exposed to the protein. The 8-anilino-1-naphthalene sulfonic acid (ANS) used in this study is a fluorescent probe sensitive to the polar groups of proteins and can interact with hydrophobic groups to generate a fluorescence spectrum, which is often used to characterise the exposure of hydrophobic groups in proteins [43]. As depicted in Figure 4, the surface hydrophobicity of Co2 extracted under strongly acidic conditions was the highest (*p <* 0.05), while the Co extracted by alkaline method was smaller, with Co10 slightly less than Co9 and Co11, similar to the studies conducted on pea [44] and tilapia [45]. It may be that the protein has a greater net positive charge and a high degree of denaturisation, which causes the advanced structure to extend and unfold, exposing aliphatic and aromatic amino acids that form the hydrophobic core and increasing surface hydrophobicity. In addition, combined with the solubility results, surface hydrophobicity was negatively correlated with the overall solubility trend, indicating that higher solubility was related to the decrease in hydrophobic groups on the surface of protein molecules.

### 3.5. Emulsifying Property

**Figure 5:**
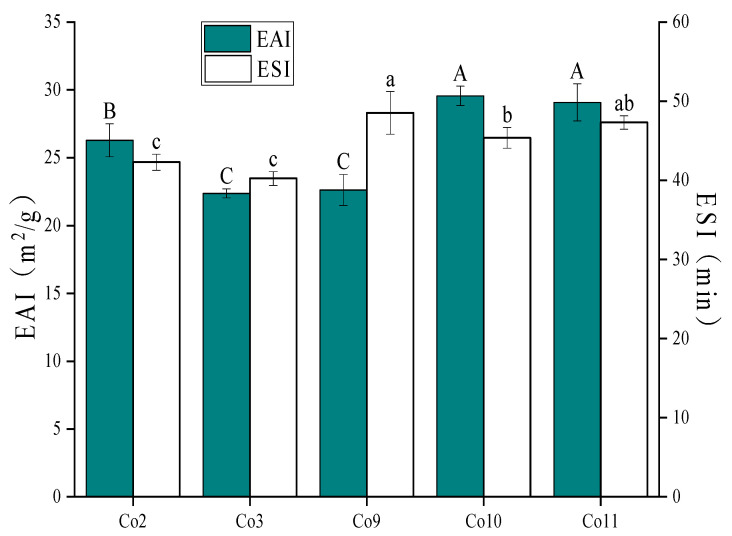
EAI and ESI of Co prepared under different extraction pH conditions. Note: EAI of different samples with different capital letters has a significant difference (*p* < 0.05), ESI of different samples with different lowercase letters has a significant difference (*p* < 0.05).

The EAI, which considers the oil/water interface area stabilised by the emulsifier per unit mass, measures the ability of proteins to resist delamination caused by the emulsion due to flocculation and coalescence to evaluate the emulsification of proteins. The emulsifying stability index (ESI) evaluates a protein’s ability to remain stable over time without phase stratification or separation. EAI and ESI are important indexes for characterising the ability of proteins to form and stabilise the oil-water interface. It can be seen from Figure 5 that the emulsifying activity of Co10 (29.56) is slightly greater than that of Co11 (29.07), while higher than that of Co2, Co3 and Co9, indicating that the emulsifying properties of Co10 and Co11 are superior, which is similar to solubility. As can be seen, solubility is a critical determinant of interfacial activity. At the same time, because vicilin is a more flexible molecule, it is easier to expand at the oil-water interface to increase the interface area [46]; the larger value of vicilin/legumin α + β may be the reason why the EAI of Co10 and Co11 is higher than that of other Co. In addition, the EAI of Co2 was significantly higher than Co3 and Co9 (*p <* 0.05), possibly due to the greater surface hydrophobicity of Co2, making it easier to adsorb to the oil-water interface. The ESI of Co9, Co10 and Co11 is better than that of Co2 and Co3 (*p <* 0.05), possibly due to the large proportion of soluble aggregates in Co9 (22.23%) and the thickness of the interface film on which Co9 is adsorbed and stable after being expanded at the oil-water interface is large, making it difficult to ‘lose stability’. It can be seen that the emulsifying activity of proteins is not necessarily related to the emulsifying stability, which is consistent with Barac’s [47] research conclusion.

**Figure 6:**
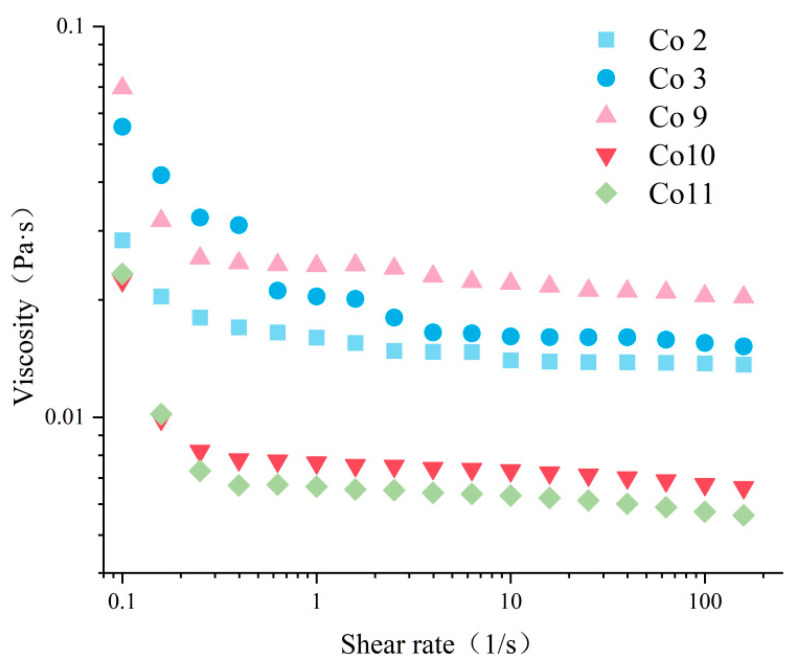
Apparent viscosity of emulsion of Co prepared under different extraction pH conditions.

**Figure 7:**
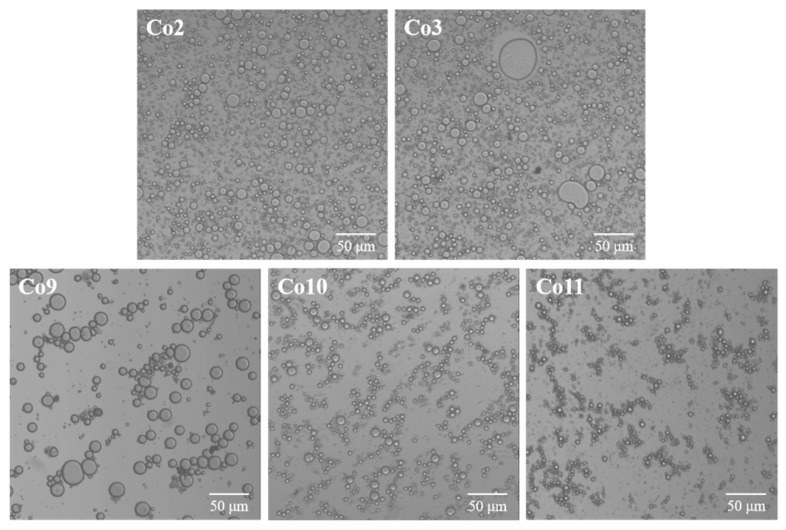
Micromorphology of an emulsion of Co prepared under different extraction pH conditions.

The apparent rheological properties of emulsions can predict their processing and storage stability, and viscosity is the main evaluation index of food emulsion taste and stability. The change in the apparent viscosity of a Co stabilised emulsion with a shear rate is shown in Figure 6. With an increase in the shear rate, the viscosity of the five Co emulsions decreased, and the phenomenon of shear-thinning was evident. This implies that the flocs formed by emulsion droplets are destroyed during the shear process and thus belong to typical non-Newtonian fluids. A similar phenomenon of emulsion shear-thinning has been reported in the literature [48]. The emulsion prepared by Co11 and Co10 has the lowest viscosity, while Co9 has the highest. According to Stokes’ law, the degree of flocculation of the emulsion is positively correlated with viscosity and shear-thinning [49]. The larger viscosity of the Co9 emulsion can be attributed to the fact that Co9 contains the most aggregates (Table 1) and the largest degree of stable oil droplet flocculation (Figure 7). This result also confirms that Co9 has the largest ESI. The apparent viscosity of Co10 and Co11 are similar, showing a trend of low viscosity and slow decline; this is related to their high ESI values and smaller oil droplet size (Figure 7). However, the viscosity of Co2 and Co3 are higher than those of Co10 and Co11, which is contrary to the ESI results; this is due to their larger particle size and higher degree of flocculation of the stabilized oil droplets.

The microstructure of the emulsion directly reflects the emulsifying properties of Co prepared by different extraction pH values, as shown in Figure 7. The average size of the Co10 and Co11 stable oil droplets is small, and the diameter of most oil droplets in Co9 is over 10 μm, but the larger oil droplets can be stabilised due to the thicker interface film. Co3 emulsion droplets distribution is uniform, but the number of protein particles that did not dissolve is greater. This will cause the oil surface protein adsorption quantity to be less, not enough to completely envelop the oil droplets, resulting in a poor interface stability that makes oil droplet coalescence more likely; this may be the cause of Co3 appearing to have larger droplets. This phenomenon is similar to the research results on the emulsion of soybean-tilapia Co [50]. Interestingly, by comparing the microscope images of the three alkali and two acid methods of Co, it is found that the acid extraction method of Co results in more round oil droplets that are more evenly distributed. However, the emulsion droplets of alkaline Co are connected in clusters, indicating that the interface adsorption mode tends to be ‘bridging adsorption’ with protein as the core, which is consistent with the result of a high proportion of large molecular weight protein and low rheological viscosity [51]. The comparison of emulsion micromorphology further confirms that alkali Co’s emulsifying properties are better than those of acid Co.

### 3.6. Gel Properties

**Figure 8:**
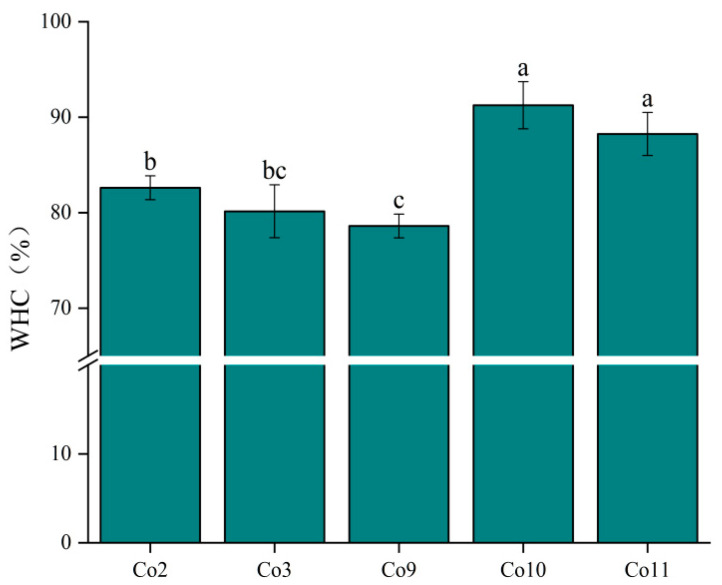
Water-holding capacity of heat-induced gel of Co prepared under different extraction pH conditions. Note: Different letters indicate that there is a significant difference between samples (*p* < 0.05).

**Figure 9:**
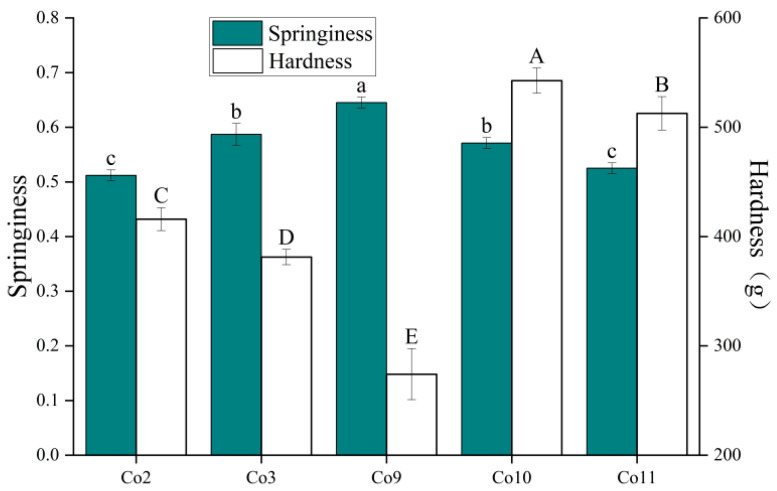
Texture properties of heat-induced gel of Co prepared under different extraction pH conditions. Note: Hardness of different samples with different capital letters has a significant difference (*p* < 0.05), and the springiness of different samples with different lowercase letters has a significant difference (*p* < 0.05).

WHC refers to the ability of the protein gel network to adsorb and lock water when subjected to external force. WHC is important for the texture and sensory properties of food, as well as an indicator of gelation quality. As shown in Figure 8, the WHC of Co10 and Co11 gels was approximately 90%, which was significantly higher than that of Co2, Co3 and Co9 (*p* < 0.05), indicating that the WHC of Co extracted under alkaline conditions is better. This is because the WHC of protein gels generally depends on the pore size and uniformity of the gel network, and gels with a fine structure are conducive to more protein–water interactions than rough gels [52], resulting in higher WHC. In addition, high gel hardness (Figure 9) makes it difficult for water molecules to escape under centrifugal conditions, while low surface hydrophobicity (Figure 4) can also improve the ability of the gel network to bind water molecules, thus improving the WHC of the protein gel.

**Figure 10:**
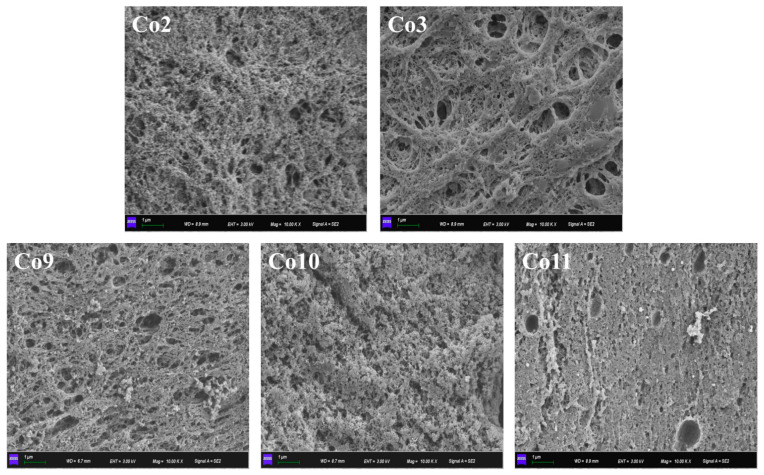
Microstructure of a heat-induced gel of Co prepared under various extraction pH conditions.

Hardness and springiness are the most important terms to evaluate the texture characteristics of solid food. Hardness denotes the maximum force required for the sample to be compressed, and springiness refers to the extent to which the gel returns to its original shape after decompression [53]. The texture test results of the five kinds of Co heat-induced protein gels are shown in Figure 9. The hardness of Co10 gel was significantly higher than that of Co11 gel (*p* < 0.05); this may be because more sulfhydryl groups are oxidised during the heat denaturation and gelation of Co10 and Co11, forming strong intermolecular forces such as disulfide bonds, which make the gel microstructure more compact and improves gel hardness. The hardness of Co2 is greater than that of Co3 (*p* < 0.05), which is due to its greater surface hydrophobicity, and which increases hydrophobic interactions during gelation, promotes protein aggregation and cross-linking and is conducive to increasing hardness. Co9 has the highest springiness and the lowest hardness (*p* < 0.05). Based on previous results, this may be due to its functional properties being more similar to plant globulins than other Co types. Therefore, during gelling, globulin enters the gel network in the form of filling rather than cross-linking (Figure 10), resulting in low hardness and high springiness.

Figure 10 shows the microstructure of five kinds of Co heat-induced gel proteins to directly illustrate the influence of different extraction pH on the gel network structure of the proteins. In general, the five types of Co protein gels were relatively dense and had fewer pores. Co2 and Co3 showed fibroid gel networks, and this loose gel structure type was not conducive to the formation of water retention and gel strength, which was consistent with the results of the WHC and hardness test and the results of the study on the thermal gel of fish myofibrin [54], indicating that Co2 and Co3 gels showed a preference to fish protein gels. The gel networks of Co9, Co10 and Co11 are more compact, perhaps because the globulin components of pea (vicilin and legumin) fill in the fibrous network as particles during gelling, resulting in a more compact gel structure [55]. The porosity of Co9 and Co11 was significantly higher than that of Co10, especially the porosity of Co11 with a diameter of 1 μm, resulting in hardness and WHC lower than that of Co10. Co10 has a coarseness similar to pea and soybean proteins, but it is denser [56]. Combined with the analysis of WHC and hardness, it could be concluded that Co10 gel demonstrated functional compatibility between pea protein and grass carp protein, that is, a flexible network structure with a certain rigidity and easy deformation and recovery, which has good application potential.

## 4. Conclusions

In this study, we investigated the protein composition, chroma, solubility, surface hydrophobicity, emulsification and gel properties of pea-grass carp co-precipitated dual-protein prepared by different extraction pH (2.0, 3.0, 9.0, 10.0 and 11.0). Overall, the functional properties of Co extracted by alkali method are better than those of Co extracted using the acid method. However, because Co9 is yellowish in colour, its application may be restricted. The functional properties of Co10 and Co11 are excellent. Co10 has the properties of both plant and animal proteins, and it outperforms Co11 in terms of vicilin/legumin α + β value, solubility, EAI, ESI, emulsion microstructure, WHC, hardness and gel microstructure. Therefore, it can be considered that the functional characteristics of Co10 have comprehensive advantages and a wide range of application values. This study establishes a theoretical foundation for future advancements in plant–animal co-precipitated dual-protein processing methodology.

## Figures and Tables

**Table 1:** The relative optical density of Co non-reduced electrophoretic bands prepared under different extraction pH conditions (%).

Sample	Aggregates	MHC	Convicilin	Leg α + β	Vicilin	AC	Leg α	TM	leg β	Others	Vicilin/Leg α + β
Co2	13.47	15.21	5.33	9.36	16.72 *		13.33	/	21.50	5.07	/
Co3	11.92	19.36	6.28	8.34	18.47 *		11.50	/	18.92	5.20	/
Co9	22.23	9.03	4.31	10.13	9.25	11.94	11.66	0.78	15.00	5.66	91.31
Co10	17.45	13.75	4.01	5.74	15.22	15.87	12.54	4.60	6.01	4.80	265.16%
Co11	20.45	16.00	3.50	5.39	13.20	13.23	13.77	2.30	4.50	7.65	244.90

Note: * indicates the total value of vicilin and AC.

**Table 2:** Chroma analysis of Co prepared under different extraction pH conditions.

Sample	L*	a*	b*	Whiteness
Co2	69.36 ± 0.32 ^c^	−0.65 ± 0.01 ^d^	11.07 ± 0.33 ^c^	67.42 ± 1.07 ^b^
Co3	67.41 ± 0.09 ^d^	−0.66 ± 0.00 ^d^	12.96 ± 0.24 ^b^	64.92 ± 0.55 ^c^
Co9	68.57 ± 0.24 ^c^	−0.47 ± 0.01 ^b^	21.23 ± 0.36 ^a^	62.07 ± 1.24 ^d^
Co10	71.23 ± 0.19 ^b^	−0.58 ± 0.02 ^c^	11.34 ± 0.29 ^c^	69.07 ± 1.47 ^a^
Co11	74.21 ± 0.24 ^a^	−0.44 ± 0.02 ^a^	12.92 ± 0.19 ^b^	71.15 ± 0.88 ^a^

Note: Different letters within the same column indicate that there is a significant difference between samples (*p* < 0.05).

## Data Availability

Data is contained within the article.
